# Challenges of implementing psychiatric rehabilitation services: a low- and middle-income country case example

**DOI:** 10.1192/bji.2021.18

**Published:** 2021-08

**Authors:** Maryam Tabatabaee, Zahra Mirsepassi, Vandad Sharifi, Yasaman Mottaghipour

**Affiliations:** Tehran University of Medical Sciences, Tehran, email: mirsepassizahra@gmail.com

**Keywords:** Rehabilitation, psychosocial interventions, implementation, low- and middle-income countries, after care services

## Abstract

This article aims to explore the barriers and challenges of implementing psychiatric rehabilitation services at a psychiatric hospital in Iran. We performed an internal mixed-methods evaluation by adopting a multi-method approach. The data were organised, analysed and interpreted by the evaluation team. A low participation rate, administrative issues, low fidelity to protocols and incomplete documentation were the main findings.

Implementing and maintaining rehabilitation services in low- and middle-income country settings requires more than a mere transfer of models of psychiatric rehabilitation; it needs adaptation to the local context as well as continuous evaluation and quality improvement in an iterative fashion, given the rapidly changing contexts with scarce resources.

More than 85% of the world population is living in low- and middle-income countries (LMICs).^1^ Those providing mental health services in LMICs are faced with several barriers, including lack of adequate infrastructure and limited financial resources, poor service delivery, scarcity of trained staff, and limited time and access.^1–4^ In this context, mental health promotion should be considered as a sociopolitical issue.^5^ Flexible policy, provision of financial resources, use of adapted evidence-based interventions, and social and cultural issues should be considered.^2^ Planning staff training, monitoring and evaluation are emphasised in the literature.^2^ Lack of sufficient evidence about the evaluation of community mental health rehabilitation and the sustainability of the services is another challenge in LMICs.^3,6^

In Iran, a low- and middle-income country, the prevalence and burden of psychiatric disorders are high. The Iranian Mental Health Survey showed that one out of every four adults aged 15–65 experienced at least one mental disorder in 12 months.^7^ Moreover, the mental health budget is not sufficient to decrease this burden.^7^

Nearly 10 years ago, a centre for providing rehabilitation and psychosocial services was established in Roozbeh hospital, which is the oldest academic psychiatric hospital in Iran and has 207 beds. The mean duration of stay is 28 days. This hospital works as one of the referral centres for patients with severe mental illness (SMI) in Tehran.

The development of the centre was described by Mirsepassi et al.^8^ The centre provides patients with SMI with telephone follow-up, patient and family psychoeducation, social skills training, cognitive rehabilitation and home care services, and recently supported employment. After a while, we decided to evaluate our services. Based on the results of the evaluation, we provided some strategies to improve the quality of the services. This article aims to briefly report the evaluation and discuss some challenges of psychiatric rehabilitation in our centre in Iran.

## Methods

We conducted an internal mixed-methods evaluation and some interventions in an iterative cycle for 2 years. The primary objective of the evaluation was to explore barriers to providing care and the quality of the services. We adopted a multi-method approach and conducted interviews with the main stakeholders, focus groups with practice staff, a document review, clinical data gathering, observation and a client satisfaction survey. The data were organised, analysed and interpreted by the evaluation and administrative team to make decisions and plan interventions.

## Results

The main evaluation findings are discussed below.

### Low participation rate

**Fig. 1:**
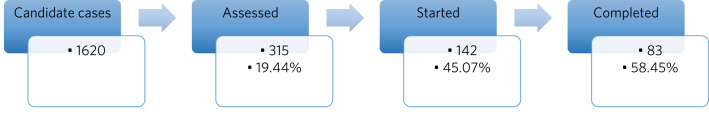
Recruitment process and participation rate of clients at Roozbeh Day Centre.

In total, 2550 patients were discharged from the hospital during the 2 years of the project; 1620 of those were candidates to receive at least one psychosocial service in the centre. More than half of these patients had bipolar disorder (*n* = 918, 56.66%). Patients who did not reside in Tehran or were diagnosed with a non-severe mental illness were not considered for our services. All candidates were invited to attend a rehabilitation assessment session. Among the 1620 candidates, 315 (19.44%) accepted our invitation for the assessment. Of these, 142 (45.07%) participated in the first session of the recommended service. When they attended the first session, the participation rate was 58.45% (83 patients) (Fig. 1).

The low participation rate could be related to four main factors: first, clinicians do not emphasise psychosocial services as one of the main components of the treatment plan, and these services are not among their priorities; second, the perceived need for non-pharmacological treatments is low among patients and their families, especially in underprivileged regions; third, a substantial proportion of patients have financial problems that cause difficulties in paying their medical expenses and even transport costs; and, fourth, patient engagement for psychosocial interventions did not happen during hospitalisation. In other words, the referral of the patients was not embedded in the routine practice. Instead, the rehabilitation centre staff reviewed the records of the patients and invited them to receive the appropriate services.

### Administrative issues

Owing to administrative and economic issues in recent years in Iran, some motivated and well-trained staff left the centre. Unexperienced and temporary staff were unable to engage patients and families during the assessment session.

### Low fidelity to protocols

We found that not following the guidelines led to problems such as missing follow-up reminders, an irregular schedule and variable length of sessions. We believe these problems contributed to the drop-out rate.

### Inaccurate and incomplete documentation

Failure to record some important data and poorly documented information caused problems in monitoring administrative processes, supervision and data interpretation.

## Discussion

Sufficient evidence suggests that psychosocial services can enhance patients’ functioning and quality of life. A review of psychoeducation in Iran shows the effectiveness of these services. However, the implementation of the services in routine clinical settings is still a challenge.^9,10^ In our centre, the low participation rate was the main problem, and drop-out from the recruitment process was prominent in two phases: first, between the number of candidates for the services and the number of participants in the assessment session; and, second, between the number of participants in the assessment session and those who started the proposed services.

### Challenges

Despite the widespread implementation of psychosocial services for patients with SMI, evidence for their feasibility in LMICs is still lacking. Brooke-Sumner et al^6^ have shown despite their acceptability, factors such as low education levels, unavailability of caregivers, and difficulty in follow-up of participants may have hindered the implementation of such services in LMICs. This is an example of the ‘implementation gap’, which refers to difficulties in translating evidence into routine practice; this gap could be even larger in LMICs.^11^ Some studies in Middle Eastern countries, including Egypt and Morocco, have specifically reported the existing gap between mental health needs and service access, the necessity to shift from hospital-based to community-based services, and insufficient resources.^12,13^

#### Administrative issues

Implementing psychosocial services and sustainability are challenges in LMICs that are affected by administrative support. The leader has a critical role in increasing staff motivation, providing an opportunity to learn from experiences and dealing with resistance to change.^14^

Moreover, improving the attitudes of policy makers and motivating them to invest in psychosocial services are proposed solutions to overcome the existing administrative issues. Representing a successful model can provide a benchmark for other centres.

#### Financial issues

In recent years, we have experienced financial crises in our country. As a result, we experienced more pressure on our limited resources and lost some motivated staff, which negatively influenced the quality of the services. Given the low socioeconomic status of most patients referred to the hospital, they usually experience difficulties in meeting their basic needs. Therefore non-pharmacologic treatments might be considered an extra-luxurious intervention. Furthermore, the insurance coverage for psychosocial services is insufficient.

To tackle these financial barriers, some solutions can be recommended, including securing funds from various sources, highlighting the role of psychosocial services in decreasing risk of readmission and the overall cost of care, advocacy to change insurance policies, and encouraging charities to support these services.

#### Attitude toward non-pharmacological treatments

As noted earlier, the attitudes of academic members, psychiatry residents and staff towards psychosocial interventions contributed to the low referral rate. The patients’ and families’ attitudes and expectations toward these treatments were a challenge. Rehabilitation services are relatively new in the country, and these treatment modalities are not part of routine practice in most mental health facilities. To improve attitudes, we should focus on integrating these services into routine care, investing in the training of healthcare providers and developing psychosocial treatments in the residency curriculum.

### Recommendations

Implementing rehabilitation services in LMICs requires more than a mere transfer of models of psychiatric rehabilitation. Given the rapidly changing contexts and scarce resources, it needs adaptation to the local context, as well as continuous evaluation and quality improvement in an iterative fashion. Continuous education of mental health professionals regarding the importance of psychosocial interventions should form part of implementation plans.^15^ Furthermore, patient and family education about the necessity of psychosocial services is recommended.

Advocacy is considered by the World Health Organization (WHO) to be one of the 11 areas for action in any mental health policy because of its benefits for consumers and families.^1^ In the context of underfunding of mental healthcare, the WHO has recently announced the goal of the 2020 World Mental Health Day campaign to increase investment in mental health programmes at the national and international levels. Advocacy efforts in LMICs should help local and governmental authorities to prioritise mental health. Patient and user groups can be encouraged to participate in these efforts, especially in limited-resource settings.^16^ As a result, insurance organisations could be persuaded to reimburse the costs of psychosocial services.

In some countries, telepsychiatry has been developed to increase access to mental health services.^2^ Recently, the COVID-19 pandemic has persuaded hospital authorities to develop telepsychiatry services. Online psychosocial services might be a potential solution.

## Conclusions

Implementation and sustainability of rehabilitation services face different challenges. Given the rapidly changing contexts and scarce resources, continuous evaluation, quality improvement, training and advocacy at different levels are recommended.
